# A Case Report of Renal Tuberculosis With Associated Unusual Pulmonary Findings

**DOI:** 10.7759/cureus.19972

**Published:** 2021-11-28

**Authors:** Abdullah Almazloum, Tasnim Elgazzar, Maha Alkhayat, Lina A Alansari, Sami Almustanyir

**Affiliations:** 1 Pulmonology Department, Prince Mohamed Bin Abdulaziz Hospital, Riyadh, SAU; 2 College of Medicine, Alfaisal University, Riyadh, SAU; 3 Infectious Diseases Department, Prince Mohammad Bin Abdul Aziz Hospital, Riyadh, SAU

**Keywords:** renal tuberculosis, pulmonary tuberculosis, kidney, focal segmental glomerulosclerosis, extrapulmonary, tuberculosis

## Abstract

Tuberculosis (TB) is a wide-reaching chronic inflammatory disease predominantly infecting the lungs. When it infects other sites, it is termed extrapulmonary TB. Among the extrapulmonary forms, genitourinary TB (GU-TB) accounts for 30%-40% of cases.

We report a case of pulmonary-renal TB with unusual pulmonary ﬁndings. Subsequent investigation of a frank haematuria case revealed positive Mycobacterium TB culture and acid-fast bacillus polymerase chain reaction (AFB-PCR) samples of urine, with abdominal imaging ﬁndings suggestive of GU-TB. Pulmonary involvement was evident on chest imaging as bilateral innumerable small nodules and tree-in-bud pattern with negative AFB-PCR from bronchoalveolar lavage samples. Clinicians practicing in endemic countries should adopt a high index of suspicion to avoid treatment delays and the development of complications of GU-TB.

## Introduction

Tuberculosis (TB) is a communicable chronic infectious and inflammatory disease caused by the bacillus Mycobacterium tuberculosis complex. Despite TB being curable and preventable, 10 million people contracted TB in 2019 and 1.4 million died from the disease in the same year [1]. TB classically affects the lungs (pulmonary TB), but it can involve other organs (extrapulmonary TB) [1]. Globally, genitourinary TB (GU-TB) is labeled as one of the rare forms of extrapulmonary TB; however, misdiagnosis and underreported cases are major issues affecting the accuracy of epidemiological data. There is an alarming absence of recent and local studies in the literature regarding renal and pulmonary TB with atypical presentations. With the aim to add to the collective literature and raise awareness of GU-TB, we present a case of PCR-positive renal TB with a radiological tree-in-bud pattern of pulmonary TB.

## Case presentation

**Figure 1:**
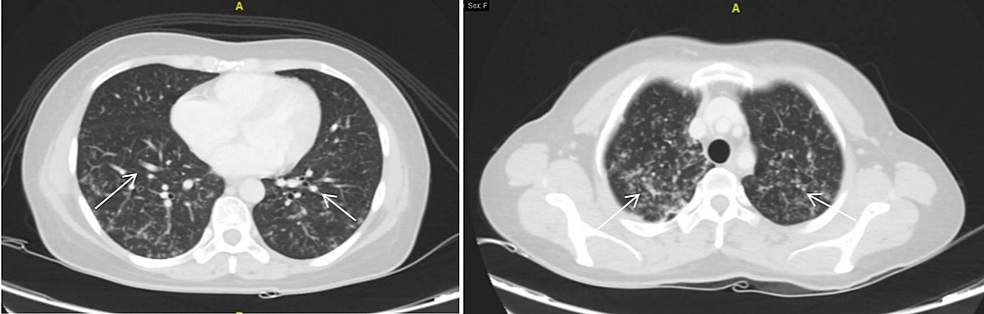
Axial CT images of the lung demonstrating diffuse tree-in-bud appearance with micro-nodularity (arrows) involving upper (left) and lower lobes (right). CT: computed tomography

**Figure 2:**
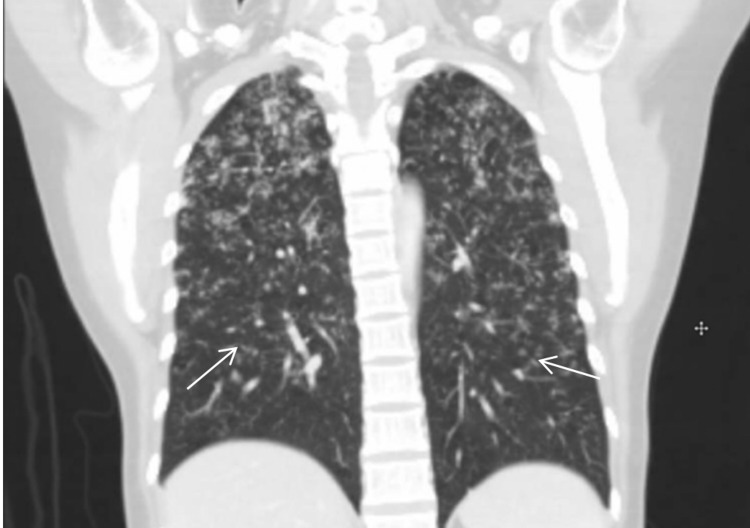
Coronal chest CT imaging showing diffuse tree-in-bud appearance with micro-nodularity involving upper and lower lobes. CT: computed tomography

**Figure 3:**
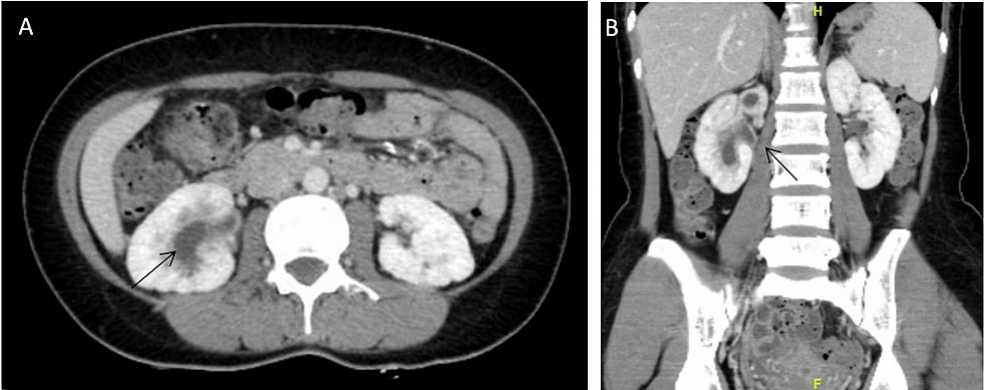
Axial (A) and coronal (B) contrast-enhanced computed tomography images of the abdomen and pelvis subsequently revealed right kidney multiple dilated calyces and renal pelvis, mild proximal dilatation of the right ureter, thickening of the renal pelvis and right ureteric wall (arrows), and mild diffuse thickening of the urinary bladder wall.

A 32-year-old Filipino woman, with no significant medical and family history, presented to our emergency department complaining of two months of frank hematuria, suprapubic pain, intermittent low-grade fever, loss of appetite and unintentional weight loss of 10 kilograms in the last three months. Symptoms like night sweats, cough, hemoptysis, breathing difficulty, flank pain, joint pain or skin rash were denied. Her physical examination was within normal limits except for mild suprapubic tenderness on abdominal examination. Her temperature was 37 °C, respiratory rate was 19 breaths/min, blood pressure 100/55 mmHg, and pulse rate was regular at 82 beats/min. Prominent lab findings included a total leukocyte count of 6,200/mm^3^, elevated erythrocyte sedimentation rate of 49 mm/hr. and C-reactive protein of 1.4 mg/dL. Urea (10.7 mmol/L) and creatinine (122 µmol/L) were mildly elevated, and creatinine normalized after proper hydration. Liver function tests were normal and autoimmune workup (RF, C4, C3, ANA, ANCA) was negative. Serology examinations revealed negative results for HIV, HBsAg and anti-HCV. Urinalysis demonstrated urinary pH 6.0, WBC > 100, RBC > 100, bacteria 2+ , protein 2+; no nitrite, leukocytes, glucose or ketones. Blood and urine cultures had no growth of pyogenic agents. Renal ultrasonography and renal colic computed tomography were done to rule out bladder or ureteric stones and showed inflammatory changes in the right kidney. A provisional diagnosis of disseminated TB was established taking into account the clinical presentation, laboratory investigations and imaging findings; therefore, the patient was admitted to our internal medicine department for workup completion. A purified protein derivative (PPD) skin test was obtained and revealed negative. Renal biopsy was taken while waiting for TB workup results. TB work-up included; lower respiratory bronchoalveolar lavage and biopsies from lung and kidneys revealed negative TB-PCR. Mycobacterium TB was positive in acid-fast bacillus polymerase chain reaction (AFB-PCR) of the urine sample, which was delayed due to unavailability of test and COVID-19 restrictions, and the diagnosis of GU-TB was confirmed. Ultrasonography of the lower abdomen showed moderate hydronephrosis and bilateral mildly increased echogenicity of renal parenchyma. Contrast-enhanced computed tomography of chest, abdomen and pelvic was performed looking for other organ involvement to guide diagnostic approach. Contrast-enhanced computed tomography of the chest revealed bilateral diffuse tree-in-bud pattern without consolidation, cavitation, pleural effusion, mediastinal adenopathy or miliary nodularity (Figures 1, 2). Contrast-enhanced computed tomography of the abdomen and pelvis subsequently revealed right dilated kidney calyces and renal pelvis, mild proximal dilatation of the right ureter, thickening of the renal pelvis and ureteric wall, and mild diffuse thickening of the urinary bladder wall. Additionally, it demonstrated multiple mildly enlarged para-aortic and retrocaval lymph nodes and multiple enlarged mesenteric lymph nodes calcifications (Figures 3A, 3B).

 

Pathological reporting of transbronchial lung biopsy showed necrotizing granuloma with negative AFB stain for TB and non-reactive PAS stain for fungi. A renal biopsy under light microscopy demonstrated focal segmental glomerulosclerosis with mild interstitial fibrosis and tubular atrophy, 2/8 glomeruli reveal global sclerosis, one of the glomeruli shows adhesion of capillary loops at the tubular outlet of the glomerulus and one artery shows focal mild intimal fibrosis. Immunofluorescence staining for IgG, IgA, IgM, C3, C1q, kappa, lambda, albumin, and fibrinogen was negative. Electron microscopic findings demonstrated mesangial electron-dense deposits and diffuse effacement of the podocytes foot process.

Treatment of TB with rifampicin, isoniazid, pyrazinamide, and ethambutol was started after establishing a diagnosis and is to be continued for six months. Upon the patient’s clinical improvement, she was discharged from the hospital after arranging for follow-up visits and educating the patient and her family.

## Discussion

As reported by WHO, 16% of the cases of TB infect extrapulmonary sites [1]. GU-TB is the second most common form (20%-30%) of extrapulmonary TB in developed countries, and the third most common in developing countries in 2019 [2-5]. Reports from Saudi Arabia estimate the annual incidence rate as 9.9 per 100,000 in 2019 [1], nevertheless, the authors noticed a two-decade gap between the latest reported renal TB case in Saudi Arabia and the current date [6].

GU-TB is further classified into the more prevalent renal TB involving urinary collecting system and renal parenchyma, and genital TB involving epididymis, testis, urethra, and prostate in males and fallopian tube, endometrium, and ovaries in females [3,4,7]. Autopsy records report an incidence of GU-TB with concurrent pulmonary TB (85%) and miliary TB (65%) [2]. Another study by Chandran et al. [7] reports 13.5% of UG-TB had concurrent pulmonary TB.

M. tuberculosis disseminates to the kidney through hematogenous or lymphatic spread from a pulmonary infection focus. The organism remains indolent in the kidney for several years before evoking signs and symptoms of the disease [2].

Renal TB patients may be asymptomatic or present with site-dependent non-specific urinary manifestations including abdominal or pelvic pain, hematuria, sterile pyuria, urinary frequency, abdominal mass [2,5,7]. A urinary tract infection is unresponsive to antibiotics being highly indicative of GU-TB [2,3]. Renal TB can be a great mimicker of multiple diseases leading to late diagnosis with complications and sequelae of the disease, such as abscess formation, stricture or fistula formation, calculus formation, localized hydronephrosis, chronic kidney disease, and non-functioning kidney [2,8,9]. Risk factors include previous TB infection, immunosuppression (HIV infection, corticosteroids or immunomodulators, transplant patients), and renal or liver disease [2]. 

Comprehensive clinical evaluation should be followed by diagnostic tests. Initial testing with a PPD skin test is the standard for determining previous TB exposure. Most urine samples evaluated by microscopy show hematuria, leukocyturia, and acidic urine, and no organisms detected as GU-TB is typically paucibacillary [3]. Although urine culture is the gold standard for diagnosing GU-TB, it has been substituted with the cost-effective and widely available Nucleic acid amplification tests (NAATs) [3]. Other assays including GeneXpert MTB/RIF and Genotype® MTBDRplus have very high sensitivity and specificity of >90% reported in several studies [3]. A study done by Altez-Fernandez et al. [4] describes the vast heterogeneity of data reporting in the literature to accurately define the sensitivity and specificity of PCR; nevertheless, they state consistent high sensitivity of Xpert MTB/RIF. WHO supports the use of Xpert MTB/RIF to improve extrapulmonary TB diagnosis [1]. A renal biopsy exhibiting granulomatous inflammation and caseous necrosis definitively confirm the diagnosis of renal TB [7]. If undetected early, the granulomatous inflammation can progressively damage the kidneys, leading to chronic intestinal nephritis, papillary necrosis, glomerulonephritis with subsequent fibrosis and scarring and chronic renal failure [2]. Abdominal computed tomography is used in conjunction with the previous tests to determine the site of biopsy, detect complications, and guide surgical management if required. Highly suggestive findings of renal TB are hypo-attenuated cortical lesions and calcifications [8,9]. In regard to concurrent renal and pulmonary TB, Pinto et al [8] report that abnormal chest radiographs were found in less than half of GU-TB cases. In our case, a pulmonary finding of tree-in-bud lesions is non-specific for pulmonary TB. Studies have reported that pulmonary TB accounts for only 28% of the cause of tree-in-bud opacities, as opposed to pulmonary apical granulomas and fibrosis being more suspicious of the disease [10,11]. The presence of diffuse tree-in-buds without other associated lung lesions such as consolidation, cavitation, pleural effusion or adenopathy is considered unusual in the context of pulmonary TB. 

The first line of management of GU-TB is similar to that of pulmonary TB, and a standard treatment course of 6 months is usually sufficient in drug-susceptible GU-TB [7]. Individualized dose adjustment is needed in renal impairment [12]. Surgical management is reserved for complications and sequelae of GU-TB or coexisting renal cell carcinoma [2].

## Conclusions

Clinicians must adopt a high index of suspicion to promptly diagnose GU-TB, particularly in patients from endemic areas or carrying risk factors. These patients are at a higher risk of disease progression with higher morbidity and mortality rates in association with delayed diagnosis. A comprehensive clinical evaluation must be supported with NAATs, tissue biopsy, and radiological imaging. A standard treatment course of 6 months is usually sufficient in drug-susceptible GU-TB.
